# Young Onset Polyarticular Tophaceous Gout: Rare and Aggressive

**DOI:** 10.7759/cureus.16954

**Published:** 2021-08-06

**Authors:** Deepshikha Rana, Zeany C Momin

**Affiliations:** 1 Pathology, University College of Medical Sciences, New Delhi, IND; 2 Pathology, Pandit Bhagwat Dayal Sharma Post Graduate Institute of Medical Sciences, Rohtak, IND

**Keywords:** gout, tophi, young onset, gouty arthritis, cytopathology, gout crystal, monosodium urate crystals

## Abstract

Gouty tophi indicate a symptom of chronic gout as a result of an accumulation of monosodium urate crystals in tissues and joints, which is the most predominantly seen form of inflammatory arthritis in the middle-aged and elderly. The rarity with which it is encountered in young adults makes this a formidable diagnostic challenge.

In this report, we describe the clinical and cytopathological features of a rare aggressive case of polyarticular tophaceous gout in a young 25-year-old man with atypical involvement of hands, elbow, ankle, and feet to emphasize the importance of considering this disease entity in the differential diagnosis of multiple soft tissue masses in this age bracket as well.

## Introduction

Gout is a disorder of uric acid metabolism prevalent in the middle-aged and elderly population, characterized by recurring episodes of inflammatory arthritis along with the formation of gouty tophi [1]. It comprises asymptomatic hyperuricemia, acute gouty arthritis, intercritical gout, and chronic tophaceous gout occurring in combinations or individually [2]. Many patients also present with subcutaneous tophi as the first presentation of gout in the absence of any gouty arthritis called gout nodulosis [3].

Chronic tophaceous gout commonly occurs 10 years or more after the first acute episode [1,4]. We report an aggravated case of young-onset chronic tophaceous gout, which was diagnosed primarily on fine needle aspiration cytology (FNAC), thus emphasizing the consequences of lack of treatment and poor control of the condition.

## Case presentation

**Figure 1:**
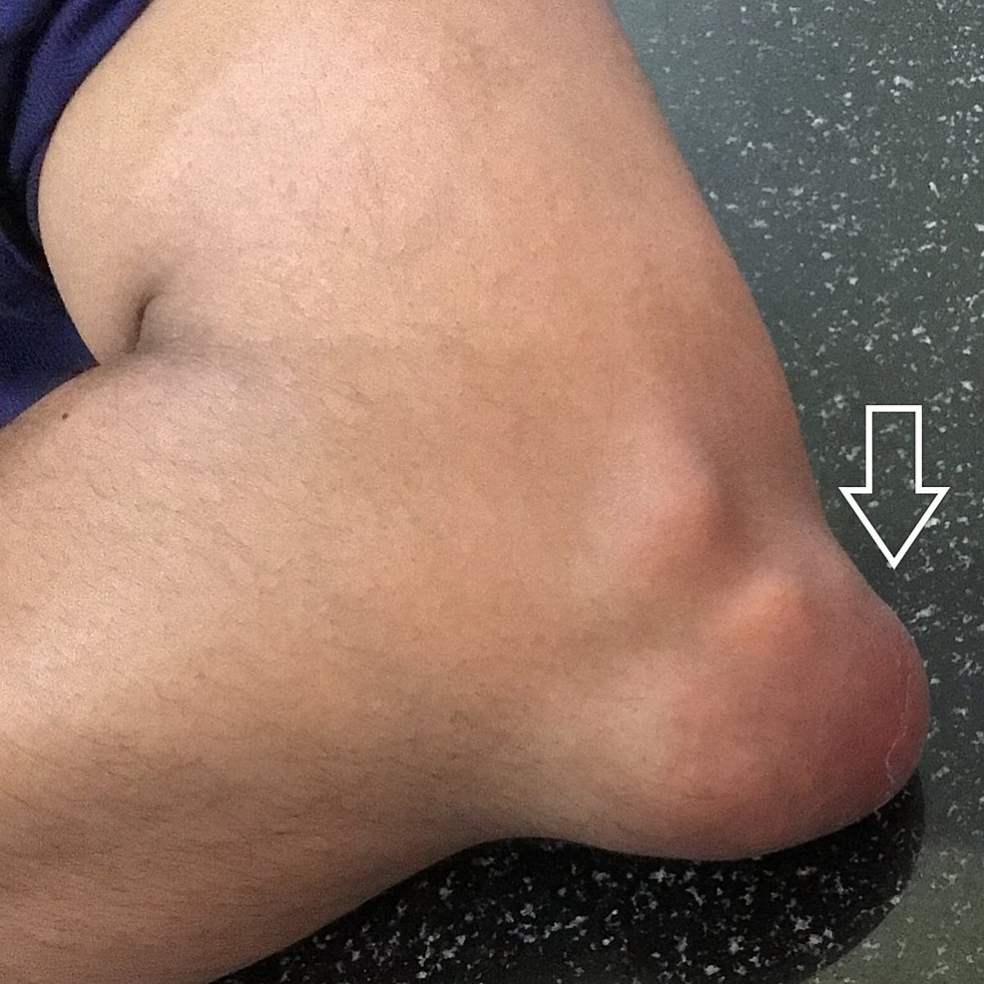
Tophi on dorsal aspect of left elbow

**Figure 2:**
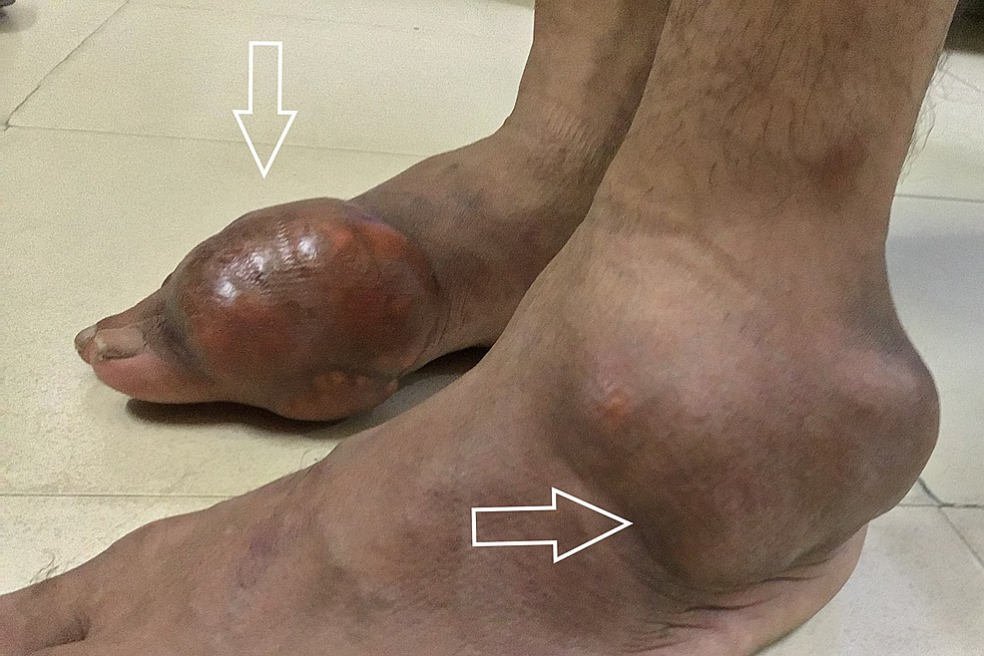
Tophi on right metatarsophalangeal joint and left lateral malleolus

**Figure 3:**
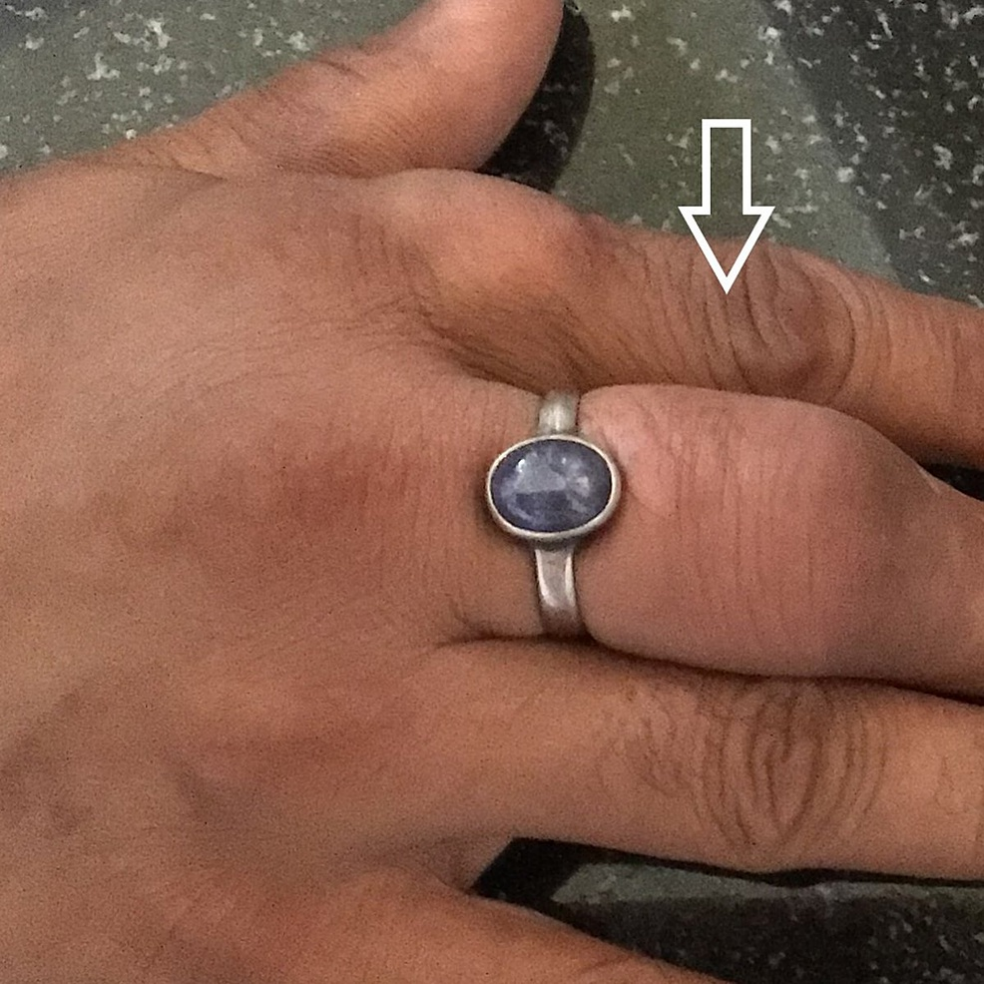
Tophi on right third interphalangeal joint

**Figure 4:**
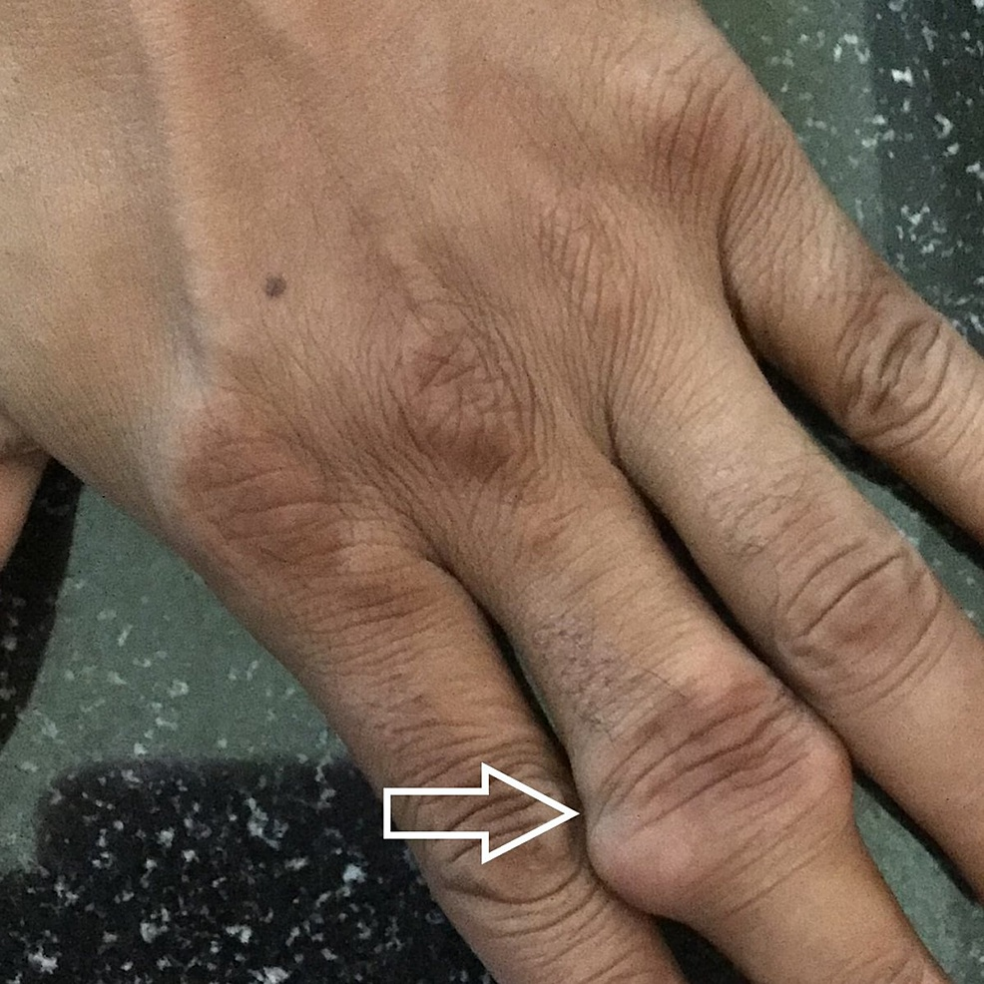
Tophi on left third interphalangeal joint

A 25-year-old male patient was referred to the cytopathology outpatient department for FNAC from swelling on the left elbow. He gave a history of trauma three years back and as a result, a clinical diagnosis of hematoma was made. Multiple swellings on the left big toe, right ankle, and third fingers of both hands were shortly noticed after the initial complaint of swelling on the left elbow. The swellings did not respond to immobilization, physical therapy, or prolonged rest nor did they subside with medications and gradually increased in size. He also complained of occasional episodes of dull aching pain which was aggravated on movement. There was no family history of joint disease and systemic illnesses. He reported occasional alcohol use and had no history of renal disease. On clinical examination, there was an approximately 5×4cm swelling over the dorsal aspect of the left elbow (Figure 1), which was well defined, immobile, firm to hard in consistency, and non-tender. Further examination revealed multiple swellings on the metatarsophalangeal joint of the right foot (Figure 2), left lateral malleolus (Figure 1) and third interphalangeal joints of both right hand (Figure 3) and left hand (Figure 4) similar to the above description.

**Figure 5:**
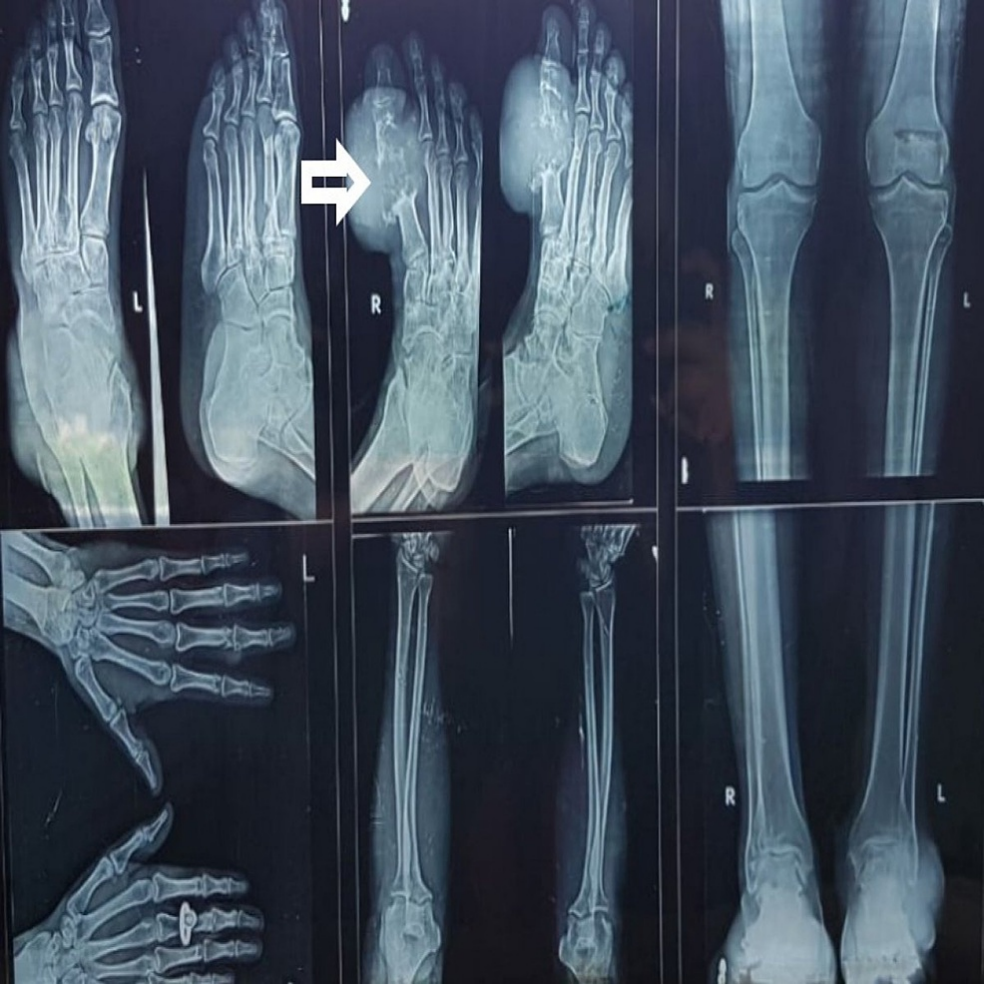
Plain radiographs of left foot, right foot, bilateral elbows left hand bilateral below knee bilateral ankle joints- revealing increased soft tissue thickness with minimal distal acoustic enhancement

Plain radiographs (Figure 5) revealed increased soft tissue thickness of the dorsal aspect of the left elbow measuring 2.4 x 1 x 1.6 cm and similar soft tissue mass in the right foot with minimal distal acoustic enhancement measuring 3.4 x 4.4 x 2.5 cm. Ultrasound and Magnetic Resonance Imaging (MRI) findings revealed large periarticular soft tissue around the first metatarsophalangeal joint involving the head and distal shaft of the first metatarsal and proximal phalanx with minimal intraarticular extension of the soft tissue causing large erosions of the head of the first metatarsal bone. In laboratory investigations, blood chemistry revealed leukocytosis 17.900/cumm, serum uric acid levels of 7.2mg/dl (normal range 3.5-7.2mg/dl), serum creatinine 1.3 mg/dL, mildly raised erythrocyte sedimentation rate (ESR), and mild anemia.

**Figure 6:**
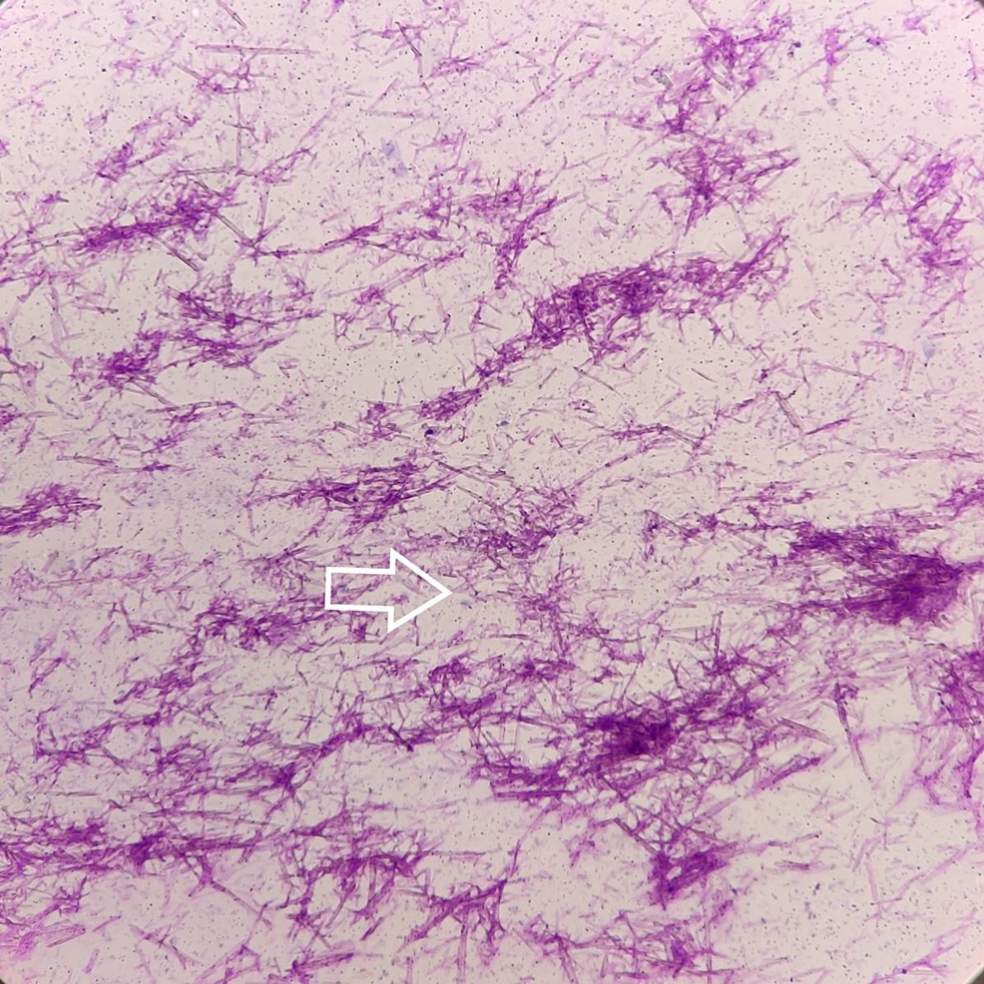
Abundant thin needle-shaped monosodium urate crystals (MSU) crystals visible under light microscope (Leishman, X400)

Fine-needle aspiration from the swellings yielded thick chalky white aspirate. Cytopathology smears prepared from the aspirate examined showed abundant small needle-shaped crystals in a blood-mixed amorphous background (Figure 6).

**Figure 7:**
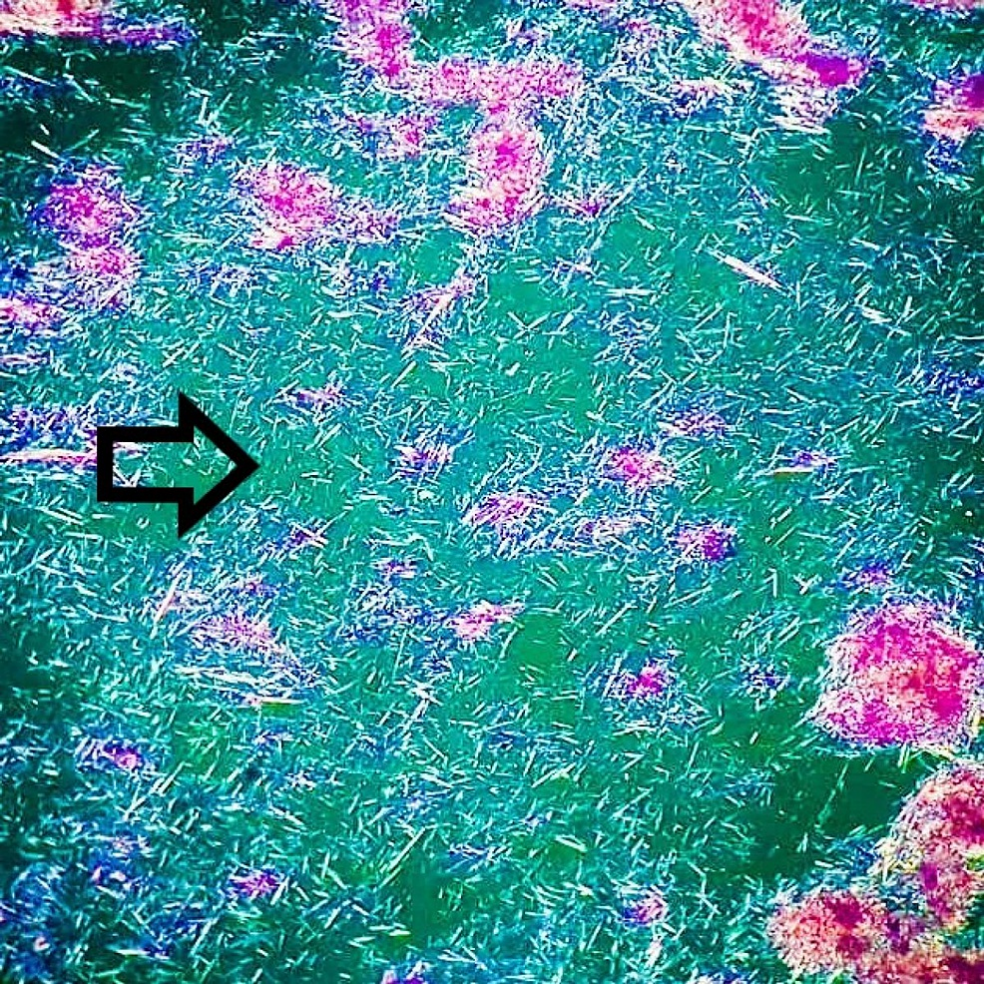
Polarizing microscopy revealing negative birefringence of stacks of needle shaped monosodium urate (MSU) crystals (Leishman, X400)

On examination of Leishman-stained cyto-smears with a polarizing microscope, the needle-shape crystals were negatively birefringent consistent with monosodium urate crystals (Figure 7).

On the basis of these findings, a diagnosis of polyarticular tophaceous gout was rendered. The patient was undergoing treatment with Department of Rheumatology on the conservative line but was lost to follow-up after few visits in the outpatient department.

## Discussion

The underlying pathology of gout was proposed by Garrod in the 18th century to be a causative relationship between elevated uric acid levels and urate crystal formation [5]. Acute episodes of gouty arthritis and tophi formation are the outcome of increased uric acid levels more than the saturation point and impaired renal uric acid excretion leading to monosodium urate (MSU) crystal deposition in joints, tendons and soft tissues [6]. In about 10% of patients, the disease may initially arise as gouty tophus [1]. Uric acid elevating drugs, genetic predisposition, other systemic illnesses and predisposing dietary factors, such as high protein diet and alcohol consumption, have been attributed to the development of gout and its symptoms [4,6-8]. However, older age, male sex, the postmenopausal state in women, and comorbidities such as renal disease, diabetes, or concomitant use of diuretics pose a higher risk [4]. Progression to chronic phase over time, painful polyarticular attacks, tophi formation in soft tissues and joints followed by complications such as disability and joint deformities are the consequences of poor control and frequent neglect of the condition despite major progress in its management [1,6,8]. Ulcerations, bone fractures, tendon, and ligament ruptures, and nerve compression syndromes can also be associated with tophi formation [8].

The differential diagnosis for gouty tophi includes chondrocalcinosis (pseudogout syndrome), a condition in which the symptoms result from diffuse deposition of calcium pyrophosphate dihydrate crystals, septic arthritis, tumoral calcinosis, synovial cysts, nodal osteoarthritis, rheumatoid arthritis, sarcoidosis, lymphoma, or neoplasms like chondrosarcoma [1,6].

Synovial fluid or tophus aspiration provides the most accurate diagnosis through visualization of needle-shaped negatively birefringent MSU crystals to differentiate from rhomboid weak positively birefringent crystals of calcium pyrophosphate dihydrate (CPPD) using polarizing microscopy and continues to be the gold standard for diagnosis of gout [1,4,7].

Management includes non-steroidal anti-inflammatory drugs (NSAIDs), allopurinol, corticosteroids, dietary and lifestyle modifications [1,4,7]. Acute attacks may sometimes resolve spontaneously without treatment and surgical treatment, although seldom required for gout, is usually reserved for cases of recurrent attacks with deformities and joint destruction [1,7,8]. Controlling hyperuricemia along with treatment of the underlying metabolic disorder, achievable with medication, is of utmost importance for reducing recurrent attacks and for the regression of tophi [1,7].

In summary, our patient presented with polyarticular tophi as an initial presentation of gout; although very rare, such cases have been reported previously in the literature [5,7]. In addition, his serum uric acid was within normal limits and was diagnosed clinically as hematoma on ultrasound in the early stages of his complaints. There were several missed opportunities to diagnose this patient earlier in his course. Gout was not considered as a part of the original differential because of its propensity to affect older individuals. Multiarticular and aggressively enlarging tophi, as exhibited in this patient, are usually noted among the older age groups who are hyperuricemic and have had repeated attacks of acute gout, often over many years.

## Conclusions

Awareness of the variable age spectrum and presentation of this metabolic disorder along with its inclusion in the differential diagnosis of soft tissue masses can aid in providing a timely diagnosis to minimize surgical intervention, joint deformities, and complications. This report highlights untreated young-onset gout complicating into multiple large tophi.
